# Unusual vitamin E profile in the oil of a wild African oil palm tree (*Elaeis guineensis* Jacq.) enhances oxidative stability of provitamin A

**DOI:** 10.3389/fpls.2024.1400852

**Published:** 2024-06-27

**Authors:** Philipp Gutbrod, Delphine Pottier, Safoora Shirvani, Katharina Gutbrod, Félicité Djien-Nyami, Raïssa Emade Ngoudjede, Georges Ngando-Ebongue, Peter Dörmann

**Affiliations:** ^1^ Institute of Molecular Physiology and Biotechnology of Plants (IMBIO), University of Bonn, Bonn, Germany; ^2^ Department of Biochemistry, Faculty of Science, University of Douala, Douala, Cameroon; ^3^ Center for Oil Palm Research (CEREPAH), Institute of Agricultural Research for Development (IRAD), Yaoundé, Cameroon

**Keywords:** mesocarp, antioxidant, palm oil, tocopherol, tocotrienol, methyltransferase, VTE4

## Abstract

**Introduction:**

The African oil palm (*Elaeis guineensis* Jacq.) is the predominant oil crop in the world. In addition to triacylglycerols, crude palm oil (CPO) extracted from the mesocarp of the fruits, contains high amounts of provitamin A (carotenes) and vitamin E (tocochromanols). Because of their unsaturated nature, the carotenes are prone to oxidation and therefore are in part limiting for the shelf life of CPO.

**Methods:**

A tree with unusual toochromanol composition was identified by HPLC screening of the mesocarp of wild trees. Polymorphisms in a candidate gene were identified by DNA sequencing. The candidate protein was heterologously expressed in Escherichia coli coli and Arabidopsis thaliana to test for enzyme activity. Oxidative stability of the CPO was studied by following carotene degradation over time.

**Results:**

In the present study, a wild Oil Palm tree (C59) from Cameroon was identified that lacks α-tocopherol and α-tocotrienol and instead accumulates the respective γ forms, suggesting that the activity of γ-tocopherol methyltransferase (VTE4) was affected. Sequencing of the *VTE4* locus in the genome of plant C59 identified a G/C polymorphism that causes the exchange of a highly conserved tryptophan at position 290 with serine. The W290S exchange renders the VTE4 enzyme inactive, as shown after expression in *Escherichia coli* and *Arabidopsis thaliana*. The oxidative stability of carotenes in the mesocarp of the wild palm C59 was enhanced compared with control accessions. Furthermore, supplementation of commercial palm oil with different tocochromanols showed that γ-tocotrienol exerts a stronger effect during the protection of carotenes against oxidation than α-tocotrienol.

**Discussion:**

Therefore, the introduction of the high γ-tocotrienol trait into elite breeding lines represents a potent strategy to protect carotenes against oxidation and extend the shelf life of CPO, hence allowing the development of a value added high-carotene CPO to be used to fight against vitamin A deficiency.

## 1 Introduction

The African oil palm (*Elaeis guineensis* Jacq.) is the most important oil crop worldwide due to its unmatched average yields of 3 to 4 tons of oil per hectare (Sambanthamurthi, 2000; Woittiez et al., 2017). Two different oils, crude palm oil from the mesocarp (CPO) and kernel oil from the kernel can be obtained from the fruits. While CPO is mainly used for edible purposes and the production of biodiesel, kernel oil is used in the oleochemical industry (Basiron, 2007). CPO is mainly composed of triacylglycerol, but also contains other important nutrients such as provitamin A (carotenes) and vitamin E (tocochromanols) (Sambanthamurthi, 2000).

Carotenes are synthesized by photosynthetic organisms such as plants, bacteria and algae but also by some non-photosynthetic bacteria and fungi (Nisar et al., 2015). Carotenes have diverse functions in humans, but the most prominent is their provitamin A property (Biesalski et al., 2007). β-Carotene which carries two β-ionone rings, is the dominant carotene in the human diet with the highest vitamin A activity (Donhowe et al., 2014). In addition to β-carotene, palm oil contains α-carotene which carries a β-ionone and an α-ionone ring, and the ratio of of β-carotene to α-carotene is highly variable between the various natural oil palm trees (Morcillo et al., 2021). Cleavage of β-carotene leads to two molecules of retinol (vitamin A) (Nisar et al., 2015). Retinol and related compounds are important for eyesight and help preventing night blindness (nyctalopia) and lack of tears (xerophthalmia). Vitamin A deficiency is a common health issue in the developing world, leading to preventable childhood blindness, afflicting thousands of malnourished children each year, with more than 100,000 deaths per year (Stevens et al., 2015). Due to its high carotene content, CPO represents an ideal, available dietary source for vitamin A in sub-saharian countries. Indeed, unlike other regions in the world where palm oil is consumed exclusively in its refined form, CPO is a major ingredient of many culinary recipes in sub-saharian Africa. However, oxidative degradation of the highly unsaturated carbon chain of carotenes in CPO during storage represents a bottle neck, leading to a rapid alteration of the quality of the oil, ultimately limiting shelf life and thus provitamin A supply.

Unsaturated lipids including fatty acids or carotenes can be stabilized by antioxidants like tocochromanols. Tocochromanols harbor vitamin E activity which is essential for human nutrition. The antioxidative activity of tocochromanols is associated with their radical scavenging capacity, because they can remove reactive oxygen species and other radicals from oils during storage. The mesocarp of African oil palm is rich in vitamin E (tocochromanols). Tocochromanols are composed of a polar chromanol ring bound to a long isoprenoid side chain which can be derived from phytol (tocopherols) or geranylgeraniol (tocotrienols) (Dörmann, 2007; Fritsche et al., 2017). Tocopherols are widespread in the plant kingdom, while tocotrienols are mostly found in monocot plants including oil palm (Cahoon et al., 2003). The number of methyl groups on the chromanol ring defines the different forms of tocochromanols, α, β, γ and δ (DellaPenna, 2005; Mène-Saffrané and DellaPenna, 2010; Fritsche et al., 2017). Tocochromanols are synthesized by conversion of p-hydroxyphenylpyruvate derived from the shikimate pathway into homogentisate which is condensed with phytyl-diphosphate or geranylgeranyl-diphosphate to form the precursors of tocopherols or tocotrienols, respectively (Cahoon et al., 2003). Cyclization by tocopherol cyclase (VTE1) gives rise to δ-tocopherol or δ-tocotrienol, respectively. Alternatively, methylation at position C3 results in the formation of intermediates which are converted by VTE1 into γ-tocopherol and γ-tocotrienol (Porfirova et al., 2002; Cheng et al., 2003). Finally, γ- and δ-tocochromanols are methylated at the position C5 by γ-tocopherol methyl transferase (γTMT/VTE4) to generate α- and β-forms of tocochromanols (Bergmüller et al., 2003). In the CPO of many accessions, γ-tocotrienol is most abundant, followed by α-tocotrienol, α-tocopherol and low amounts of δ-tocotrienol (Choo et al., 1996). However, there are several genotypes enriched with α-tocopherol and α-tocotrienol, as the VTE4 activity (% of α-forms) can vary from 25 to 75% within the species (Luo et al., 2020; Morcillo et al., 2021). The VTE4 protein is localized to chloroplasts in Arabidopsis, along with the other enzymes of tocochromanol synthesis (Mehrshahi et al., 2013).

α-Tocopherol is the most active form of vitamin E in the human body, while γ-tocopherol shows lower activity. On the other hand, γ-tocopherol harbors higher antioxidant capacity than α-tocopherol (Olcott and Emerson, 1937). It has previously been shown that tocochromanols can protect unsaturated fatty acids and carotenes against oxidative degradation (Kamal-Eldin and Appelqvist, 1996). Therefore, the content and composition of tocochromanols are important for the oxidative stability of CPO (Schroeder et al., 2006).The antioxidant capacity of the tocochromanols differs because γ-tocochromanols are stronger antioxidants than the α forms (Olcott and Emerson, 1937; Schroeder et al., 2006).

In a screening program for CPOs with altered tocochromanol content or composition in a population of wild oil palm accessions from Cameroon, we identified a unique palm tree that accumulates high amounts of γ-tocochromanols instead of α-tocochromanols in the mesocarp. We present here the molecular characterization including the identification of the *VTE4* gene responsible for the altered tocochromanol composition. Comparison of the sequences of the open reading frames of *VTE4* in this palm tree with that in the elite line Deli x La Mé revealed a single nuclear polymorphism (SNP) which gives rise to a non-conserved amino acid exchange. Expression of the *VTE4* sequence from the high γ-tocochromanol line in *Escherichia coli* and in the *Arabidopsis vte4-1* mutant deficient in α-tocopherol revealed that this SNP is responsible for the loss of VTE4 activity. Furthermore, we demonstrate that the accumulation of γ-tocochromanols in the CPO provides an improved protection of carotenes against oxidative degradation. We envision that the introduction of related traits into elite breeding material will allow for the generation of oil palms which produce a CPO with improved oxidative stability.

## 2 Materials and methods

### 2.1 Plants and growth conditions

Seeds of wild trees of the African oil palm (*Elaeis guineensis* Jacq.) growing on different sites of the natural palm grove in Cameroon were collected and germinated at the nursery in La Dibamba (Institute of Agricultural Research for Development, Center for Oil Palm Research, IRAD-CEREPAH; **Supplementary Table 1**). Wild trees and control trees of the Deli x La Mé elite hybrid were planted in the field in 2011 and grown under the same conditions. Fruits of ripe bunches from these trees were harvested, flash frozen and the exocarp pealed. Leaves were also harvested and flash frozen in liquid nitrogen. The frozen mesocarp and leaf material were lyophilized.

*Arabidopsis thaliana* wild type Col-0 and *vte4-1* mutant plants (Bergmüller et al., 2003) were germinated on Murashige and Skoog medium containing 2% sucrose (Murashige and Skoog, 1962). After two weeks, plants were transferred to fresh plates, or to pots containing soil/vermiculite (2:1). Plants were grown under a 16 h light regime with light intensity of 120 µmol m^-2^ s^-1^ and 60% humidity.

### 2.2 Tocochromanol and carotene measurements in oil palm mesocarp

The lyophilized mesocarp or leaf material were homogenized in 300 mM ammonium acetate with a Precellys homogenizer (Bertin, Frankfurt). After addition of internal standards (1 µg tocol, 3 µg canthaxanthin), tocochromanols and carotenes were extracted with hexane. Tocochromanols were separated on a LiChrospher diol column (Knauer, Berlin) by isocratic elution with hexane/tertiary butylmethylether (96:4, v/v) and measured with a fluorescence detector on an Agilent 1200 HPLC system (Agilent, Waldbronn) (Zbierzak et al., 2010). After a 1:10 dilution with methanol/tertiary butylmethylether (89:11), carotenes were separated on an EC Nucleoshell column (Macherey & Nagel, Düren) by isocratic elution with methanol and measured with an diode array detector using an Agilent 1100 HPLC system (Thayer and Björkman, 1990; Jeffrey et al., 1997).

### 2.3 Sequence analysis of EgVTE4 from palm tree C59

Genomic DNA was extracted from lyophilzed mesocarp of Deli x La Mé and of palm C59. Exons and flanking intron sequences of the locus LOC105033221 which shows sequence similarity to *Arabidopsis* AtVTE4 (At1g64970) were amplified by PCR using the oligonucleotides Bn3839, Bn3840, Bn3841, Bn3842, Bn3843, Bn3844, Bn3845 and Bn3846 (**Supplementary Table 2**), and the PCR products were sequenced by Sanger sequencing.

The plastid transit peptide of EgVTE4 was predicted using ChloroP1.1 and TargetP 2.0 (cbs.dtu.dk/services/TargetP/) (Emanuelsson et al., 1999; Almagro Armenteros et al., 2019). VTE4 protein sequences from different plant species were retrieved at UniProt (uniprot.org). Sequences were aligned using T-coffee (Notredame et al., 2000) and illustrated with boxshade (embnet.vital-it.ch/software/BOX_form.html). The domain structure of AtVTE4 was analyzed using pfam (pfam.xfam.org) (Mistry et al., 2021). The AtVTE4 sequence (At1g64970) was used for homology-modeling of the three-dimensional structure at Swiss-Model (https://swissmodel.expasy.org/) (Waterhouse et al., 2018).

### 2.4 Expression of EgVTE4 in *E. coli* and tocochromanol supplementation

For expression in *E. coli*, mature EgVTE4 lacking the transit peptide was amplified with Bn3902 including a 6xHis tag, and Bn3896 (**Supplementary Table 1**) from the construct pBinGG-EgVTE4 (see below). The G/C mutation was introduced into the mature EgVTE4-W290S sequence by site directed mutagenesis. Bn3902 and Bn3898 were used to amplify the 5´ fragment, and Bn3899 and Bn3896 for the 3´ fragment from pBinGG-EgVTE4. The cDNA of mature EgVTE4 and the two fragments representing mature EgVTE4-W290S were cloned into the *E. coli* expression vector pTVGG by Golden Gate assembly. *E. coli* BL21(AI) cells harboring the empty vector (EV, pTVGG), pTV-EgVTE4 or pTV-EgVTE4-W290S constructs were grown in Terrific Broth containing kanamycin. Protein expression was induced with isopropyl β-D-1-thiogalactopyranoside (IPTG) and 0.1% arabinose, and cells cultivated at 16°C overnight.

For tocochromanol supplementation, *E. coli* cells were collected by centrifugation and re-suspended in Terrific Broth with kanamycin. Tocochromanols (γ-tocopherol, δ-tocopherol, γ-tocotrienol or δ-tocotrienol) dissolved in ethanol were added and the cells incubated for 3 hours at 30°C. The cultures were diluted to the same OD_600_ values, and cells from the same culture volumes harvested by centrifugation. After washing with water, tocochromanols were extracted with chloroform/methanol (2:1) in the presence of tocol. After addition of 300 mM ammonium acetate and vortexing, the chloroform phase was harvested. The chloroform was evaporated under a stream of nitrogen and the tocochromanols dissolved in hexane for HPLC measurements.

### 2.5 Protein extraction from *E. coli* cells, polyacrylamide gel electrophoresis, western blot and γ-tocopherol methyltransferase assay

Protein was extracted from *E. coli* cells expressing EgVTE4 or EgVTE4-W290S after re-suspension in 1.5 ml lysis buffer (10 mM HEPES pH 7.8, 5 mM dithiothreitol, 240 mM sorbitol) with 150 µl 1.5 mM phenylmethylsulfonyl fluoride (PMSF). Cells were homogenized in a Precellys homogenizer. After addition of Triton X-100 (final concentration, 0.1%), samples were homogenized and protein collected in the supernatant after centrifugation at 14,000 x g. Proteins were separated by SDS polyacrylamide gel electrophoresis and stained with Coomassie Brilliant Blue R-250, or SDS gels were blotted onto nitrocellulose membranes. The EgVTE4 and EgVTE4-W290S proteins were detected with Ni-horseradish peroxidase conjugate (Kirkegaard & Perry, Gaithersburg) and the signals visualized by chemiluminescence using the SuperSignal West Pico Chemiluminescent Substrate (Thermo Scientific).

The VTE4 enzyme assay contained 200 µl 125 mM Tricine-NaOH (pH 7.6), 100 µl 1.25 mM sorbitol, 20 µl 250 mM ascorbate, 15 µl 4.6 mM S-adenosyl-methionine (SAM), 10 µl 50 mM MgCl_2_, 20 µl of different tocochromanols (1 mg/ml in ethanol) and 300 µg of *E. coli* protein (Bergmüller et al., 2003). The assays were incubated for 4 h at 25°C, and tocochromanols extracted with chloroform/methanol (2:1) in the presence of tocol. After addition of 300 mM ammonium acetate and mixing, the chloroform phase was harvested and evaporated under nitrogen. Tocochromanols were dissolved in hexane and quantified by HPLC.

### 2.6 Expression of EgVTE4 in *Arabidopsis*

The full length EgVTE4 cDNA was amplified by rt-PCR from mesocarp of Deli x La Mé using primers Bn3895 and Bn3896. The amplicon was cloned into the binary vector pTVGG carrying a 35S promoter by Golden Gate assembly. The mutation G869C at position 899 of the full-length open reading frame was introduced by site directed mutagenesis to generate EgVTE4-W290S. To this end, the oligonucleotides Bn3895 and Bn3898 were used to amplify the 5’ fragment of EgVTE4-W290S and Bn3899 and Bn3896 for the 3’ fragment. The two fragments were assembled into the vector pBinGG by Golden Gate cloning. The constructs eV (empty vector, pBinGG), pBinGG*-*EgVTE4 and pBinGG-EgVTE4-W290S were transferred into *Agrobacterium tumefaciens* GV3101-pMP90. *Arabidopsis vte4-1* plants were transformed by floral dip with *Agrobacterium* harboring one of the constructs. Transformed seeds were selected based on their red fluorescence derived from the DsRed marker and transgenic plants grown on soil. Frozen leaves were homogenized and tocochromanols extracted with diethyl ether in the presence of tocol. After addition of 300 mM ammonium acetate, the diethyl ether phase was collected and dried under nitrogen. Tocochromanols were dissolved in hexane and measured by HPLC.

### 2.7 Assay for oxidative stability of carotenes

Lyophilized mesocarp slices of Deli x La Mé (palm C10) or of the wild palms C52, C57 and C59 were ground to a powder under liquid nitrogen. The samples were placed into open tubes in an incubator at 37°C under air (oxidative conditions) for 1 to 6 weeks. Control samples (T_0_) were immediately collected. To terminate the incubation, the sample tubes were flushed with nitrogen gas to remove oxygen, closed with a cap, frozen in liquid nitrogen and stored at -80°C.

Aliquots of commercial palm oil (100% pure unrefined palm oil; country of origin, Sri Lanka; KTC Ltd., UK), (20 mg each) were dissolved in hexane and distributed to several microfuge tubes. Different tocochromanols (α-tocotrienol or γ-tocotrienol, dissolved in ethanol) were supplemented to each tube at a final concentration of 2 µg/mg of oil. All tubes were mixed and the solvents evaporated under nitrogen gas. T_0_ samples were immediately collected. The open tubes with the oil were placed at 37°C for 3 and 6 weeks as described above. Carotenes were extracted from the mesocarp powder or from the palm oil with hexane in the presence of canthaxanthin and measured by HPLC.

### 2.8 Statistical analysis

Statistical analyses were carried out using Excel (Microsoft) or SigmaPlot (Systat). Tukey HSD tests or Student t-tests were performed for statistical significance, where significant differences are denoted by different letters, or by asterisks (*, P < 0.05; **, P < 0.01).

## 3 Results

### 3.1 Identification of an African oil palm tree with altered tocochromanol profile

**Figure 1:**
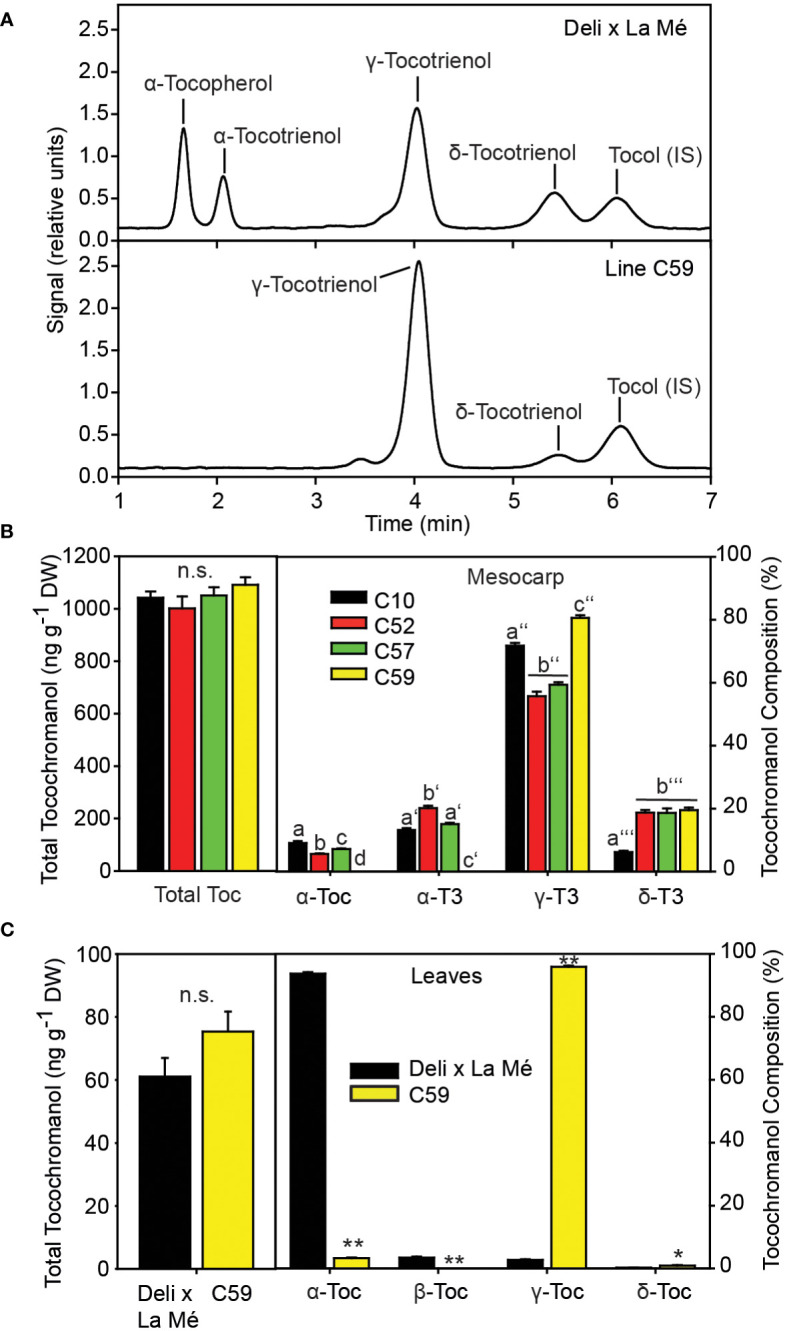
The wild oil palm C59 is deficient in α-tocopherol and α-tocotrienol. **(A)** HPLC chromatograms of tocochromanol from mesocarp extracts of Deli x La Mé and palm C59. **(B)** Tochochromanol content (left) and composition (right panel) in the mesocarp of Deli x La Mé (C10) and three wild palms (C52, C57, C59). One-way ANOVA and Tukey HSD. Different letters indicate significant differences within one tocochromanol compound. Mean ± SD; n=3; p<0.01; n.s., not significant. **(C)** Tocochromanol content (left) and composition (right) in leaves. Mean ± SD; n=3. significantly different to Deli x La Mé; t-test *p < 0.05; **p < 0.01.

Based on the previous findings that tocochromanol composition in the CPO can considerably vary, we measured the tocochromanol composition of fruits from wild trees originating from different regions in Cameroon by HPLC (**Figure 1**). One palm (C59) was identified with a unique tocochramanol profile. Palm C59 is deficient in α-tocopherol and α-tocotrienol, while it contains elevated amounts of γ-tocotrienol and δ-tocotrienol, compared with Deli x La Mé control and two other wild trees from Cameroon (**Figure 1**). The total tocochromanol contents of the mesocarp of the Deli x La Mé hybrid (C10) and of the wild palms (C52, C57 and C59) were similar. In addition, tocochromanols were measured in leaves of the four palm trees. In contrast to mesocarp, leaves of Deli x La Mé are devoid of tocotrienols and accumulate mostly α-tocopherol. However, the α-tocopehrol content in leaves of plant C59 was very low, while at the same time, γ-tocopherol accumulated, in line with the results observed in the mesocarp.

### 3.2 The Oil Palm C59 carries a G-to-C polymorphism in the genomic locus LOC105033221, causing a W to S exchange in γ-tocopherol methyltransferase

**Figure 2:**
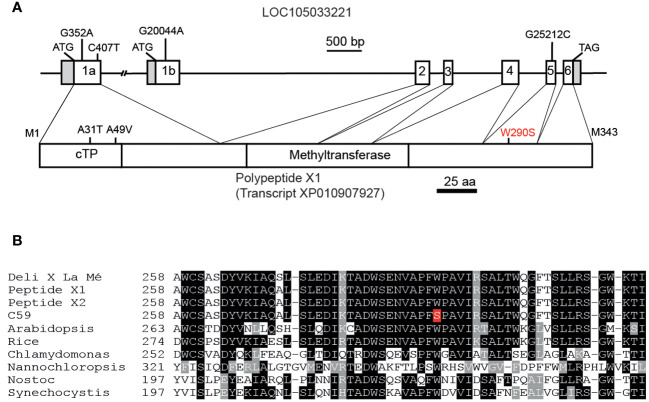
Sequence analysis of the *VTE4* gene from oil palm. **(A)** Genetic structure of the locus LOC105033221 with sequence similarity to *Arabidopsis* AtVTE4. Boxes, exons; white boxes, coding sequence; grey boxes, non-coding sequences; lines, introns. The start and stop codons are indicated. The four SNPs between the published sequence of oil palm and of palm C59 in exons 1a, 1b and 5 are indicated. The structure of polypeptide X1 derived from transcript XP010907927. The amino acid exchanges originating from the SNPs in palm C59 are indicate. The predicted chloroplast targeting sequence (cTP), the core methyltransferase domain and the SAM-dependent methyltransferase domain (pfam08241) are shown. **(B)** Alignment of amino acid sequences of VTE4 proteins from different plants, algae and cyanobacteria around the position of W290 in EgVTE4 (depicted in red). Black and grey boxes indicate identical and similar amino acids, respectively.

Because of the accumulation of γ forms and the lack of α forms of tocochromanols, the palm tree C59 presumably harbors decreased VTE4 activity, and it was possible that the *VTE4* gene in C59 carries a SNP compared with Deli x La Mé. The BLAST search with the genomic sequence of *Arabidopsis* AtVTE4 (At1g64970) in the genome of African oil palm at NCBI resulted in the identification of one locus, LOC105033221 (Ong et al., 2020). Two further loci, LOC105042568 and LOC10504611, that were previously designated EgTMT-2 and EgTMT-3 (Dou et al., 2021), displayed lower similarity with AtVTE4 and are likely not involved in tocopherol synthesis. Two transcripts have been predicted for the locus LOC105033221 which originate from alternative splicing (www.ncbi.nlm.nih.gov). Transcript X1 (XM_010907927) is derived from exons 1a, 2, 3, 4, 5 and 6, while transcript X2 (XM_010907928) is derived from exons 1b, 2, 3, 4, 5 and 6 (**Figure 2A**). The intron between exons 1a and 2 is very large (> 18 kbp), while the intron between exon 1b and 2 is ~3.5 kbp.

The subcellular localization of the two polypeptide sequences X1 and X2 was studied using the prediction tools ChloroP and TargetP. While the polypeptide X1 is predicted to be localized in the chloroplast with a chloroplast targeting sequence of 48 amino acids, the polypeptide X2 was not predicted to be chloroplast localized. Because the EgVTE4 protein likely resides in the chloroplast, the sequence X1 was considered to be the most relevant *VTE4* transcript in oil palm.

Exons and flanking intron regions of locus LOC105033221 of the Deli x La Mé hybrid and of palm C59 were amplified by PCR and sequenced. We identified four single nucleotide polymorphisms (SNPs) in the coding region between palm C59 and the Deli x La Mé/published sequence. The SNPs G352A and C407T in exon 1a result in amino acid exchanges of A31T and A49V of the polypeptide X1, while the SNP G20044A in exon 1b causes an R28Q amino acid exchange in the polypeptide X2. In addition, a G25212C exchange was detected in exon 5, which gives rise to an amino acid exchange of W290S in polypeptide X1 (or W274S in polypeptide X2) (**Figure 2A**). The amino acid exchanges of A31T and A49V in polypeptide X1, as well as R28Q in polypeptide X2 reside within the chloroplast transit sequence of X1, or at the N-terminal region of X2, and therefore presumably do not affect enzymatic activity. In addition, these amino acid exchanges do not affect the prediction for subcellular localization of X1.

The published VTE4 sequence of African oil palm, of Deli x La Mé and palm C59 were aligned with VTE4 sequences from other monocotyledoneous and dicotyledoneous plants, algae and cyanobacteria (**Figure 2B**, **Supplementary Figure 1**). The comparison of amino acid sequences revealed that the tryptophan at position 290 of EgVTE4 is absolutely conserved in the VTE4 sequences from oil palm and all other species. Therefore, the amino acid exchange W290S was a strong candidate for the cause of the deficiency in VTE4 activity in palm tree C59.

### 3.3 The W290S exchange of EgVTE4 is located in an α-helix close to the putative substrate binding site

**Figure 3:**
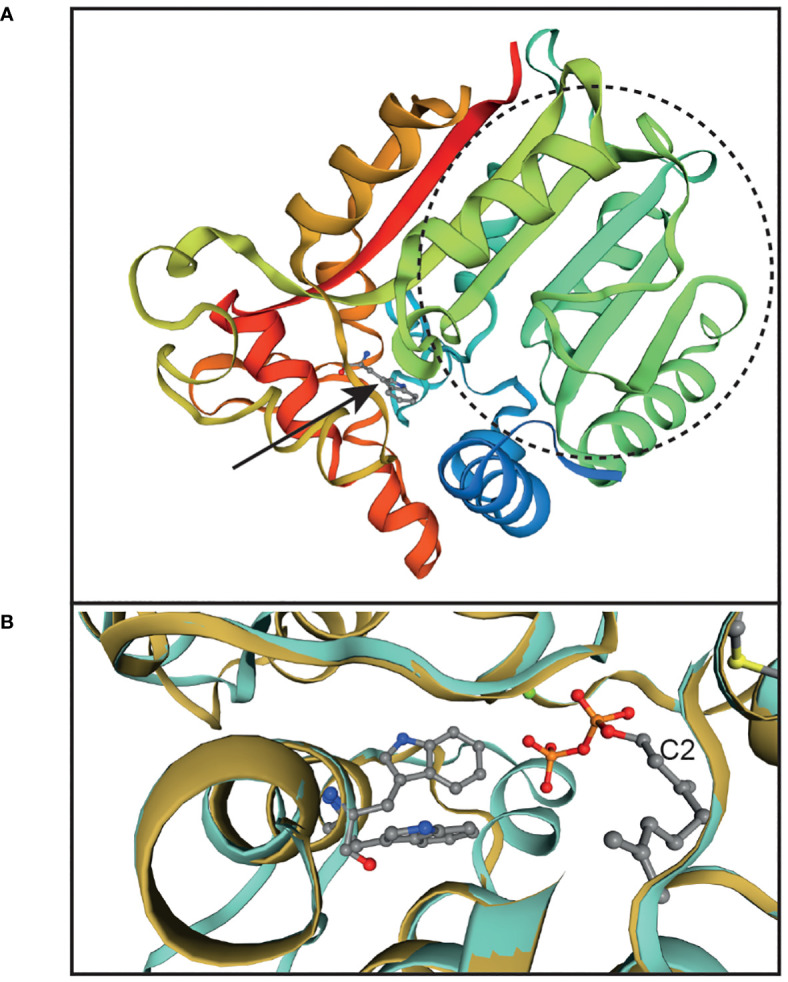
Modeling of the AtVTE4 structure indicates that the conserved W295 of AtVTE4 is in proximity with the substrate binding site. **(A)** The three-dimensional structure of the *Arabidopsis* AtVTE4 protein (At1g64970, mature polypeptide from amino acids 51-348 without chloroplast targeting peptide) was modeled according to the structure of the *Streptomyces coelicolor* GPP-MT protein using Swiss-Model. The region of the methyltransferase domain of AtVTE4 (pfam08241, amino acids 131- 229) is circled and depicted in green to turquoise color. The arrow points to W295 of AtVTE4 (equivalent to W290 of EgVTE4) which is shown as a ball-and-stick structure. **(B)** Overlay of the two structures of AtVTE4 (turquoise) and *Streptomyces* GPP-MT (gold). The close-up shows the area around W295 in AtVTE4 (top ball-and-stick structure) and the corresponding tryptophan in GPP-MT (bottom). The C2 of geranyl-diphosphate, the substrate of GPP-MT, is indicated. The sulfur of S-adenosyl-homocysteine, the methyl donor, is shown in yellow in the top right corner.

Analysis of the domain structures of the *Arabidopsis* AtVTE4 protein (Q9ZSK1) at EMBL (http://pfam.xfam.org/) revealed the presence of a conserved γ-tocopherol methyltransferase region (accession PLN02244) which includes the S-adenosyl-methionine (SAM)-dependent methyltransferase domain (pfam08241) at positions 131-229. For three-dimensional modeling of the structure, we used the AtVTE4 sequence to search for related sequences at the Swiss-Model web page. The geranyl-diphosphate 2-C-methyltransferase (GPP-MT) was retrieved as the most similar sequence with identity of 23.5%. This enzyme catalyzes the methylation of geranyl-diphosphate at the C2 position and is involved in the synthesis of the terpenoid 2-methylisoborneol in *Streptomyces coelicolor* (Köksal et al., 2012). The structure of GPP-MT has been elucidated with its substrate and cofactors, i.e. Mg^2+^, S-adenosyl-L-homocysteine (methyl donor) and geranyl-diphosphate at 2.05 Å resolution (PDB file 3vc2.1.pdb). The GPP-MT structure was used for the modeling of the AtVTE4 sequence using Swiss-Model (**Figure 3A**). The green and turquoise sequence parts correspond to the methyltransferase domain (pfam08241). The position of W295 of AtVTE4 (equivalent to W290 in EgVTE4) is located in another α-helix outside of this domain. The two structures of GPP-MT (3vc2.1.pdb) and AtVTE4 (model_01.pdb) were overlaid using the structure comparison module of Swiss-Model. **Figure 3B** shows that the tryptophan at position 295 of AtVTE4 and the corresponding tryptophan of GPP-MT localize in the same α-helix, and that they extend into a cavity close to the substrate of GPP-MT, i.e. geranyl-diphosphate. Therefore, it is likely that the tryptophan 295 in AtVTE4 is crucial to mediate substrate binding for methylation.

### 3.4 The G-to-C polymorphism in EgVTE4 of palm C59 abolishes VTE4 activity after expression in *Escherichia coli* or *Arabidopsis*

**Figure 4:**
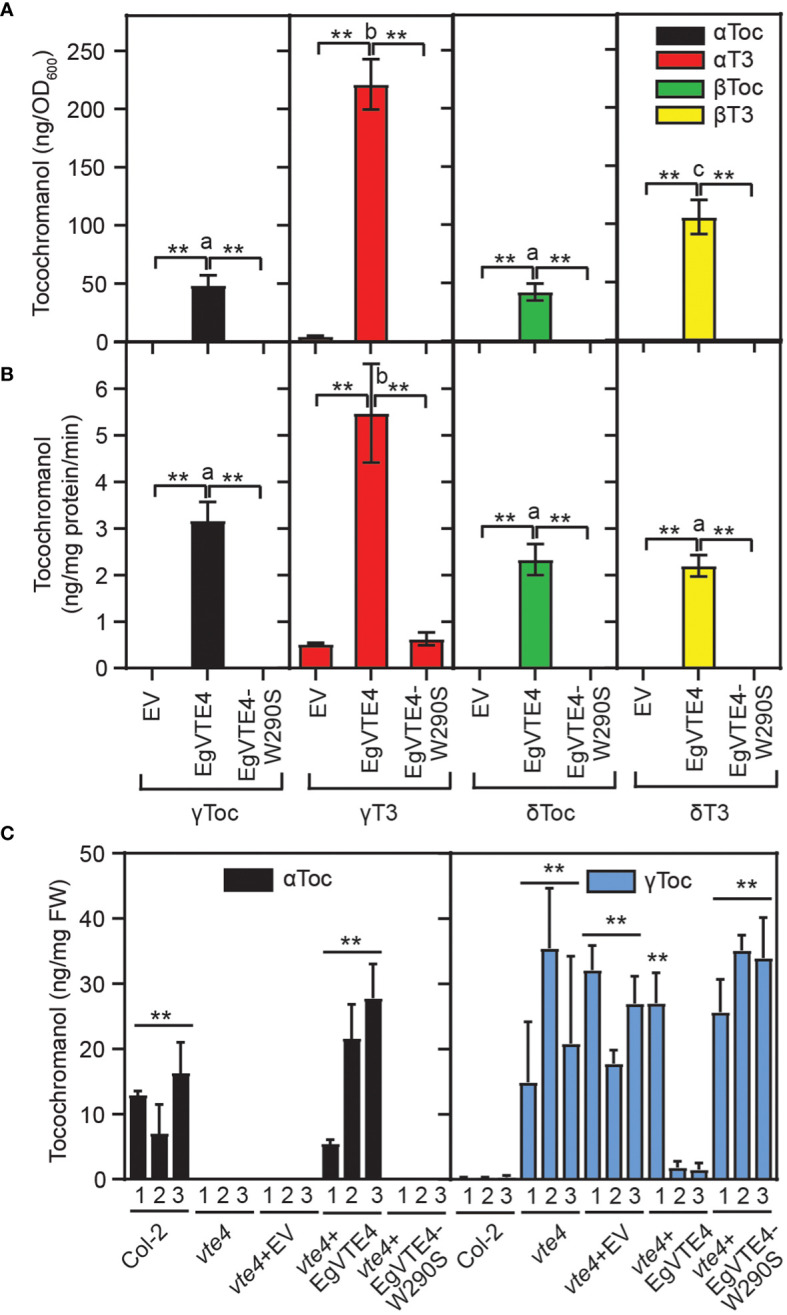
The C to G mutation of EgVTE4 results in loss of γ-TMT activity. **(A)** *E. coli* cells expressing EgVTE4 or EgVTE4-W290S were supplemented with different tocochromanols. After lipid extraction, tocochromanols were measured by HPLC. **(B)** EgVTE4 and EgVTE4-W290S proteins were isolated from recombinant *E. coli* cells and used for *in vitro* assays of VTE4 activity with different tocochromanols and SAM. In **(A, B)**, T test was used to analyze differences between activities of EgVTE4 vs. control (EV) and EgVTE4-W290S cells in experiments with the same tocochromaonol (**p<0.01). Significance of differences between activities of EgVTE4 with different tocochromanols was tested by one-way ANOVA and Tukey HSD (different letters indicate significant differences, p<0.01). Mean ± SD; n=3. **(C)** The full-length EgVTE4 and EgVTE4-W290S mutant proteins including the predicted chloroplast targeting sequences under control of the 35S promoter were introduced into the *Arabidopsis vte4-1* mutant. Tocochromanols in the leaves of three independent plants per line were measured by HPLC. T test. Mean ± SD; n=3; p<0.01. ** indicates significant differences of Col-2 and *vte4*+EgVTE4 vs. *vte4*, *vte4*+EV and *vte4*+VTE4-W290S. Note that plant 1 of vte4+VTE4 has significantly higher γ-tocopherol contents compared with plants 2 and 3. αToc, α-tocopherol; αT3, α-tocotrienol; βToc, β-tocopherol; βT3, β-tocotrienol; γToc, γ-tocopherol; γT3, γ-tocotrienol; δToc, δ-tocopherol; δT3, δ-tocotrienol.

To unravel whether the polymorphism of EgVTE4-W290S in palm C59 results in a loss of VTE4 activity, the cDNA of the mature form of the EgVTE4 sequence X1 lacking the predicted transit peptide was obtained from Deli x La Mé. A mutant version of EgVTE4, EgVTE4-W290S, carrying the G-to-C polymorphism was created by site directed mutagenesis. The two sequences were expressed in *E. coli* cells. Separation by SDS polyacrylamide gel electrophoresis and Western blotting revealed that the two proteins EgVTE4 and EgVTE4-W290S were expressed at similar levels with sizes of ~34 kDa close to the calculated sizes (33.1 kDa) (**Supplementary Figure 2**). Therefore, the amino acid exchange W290S does not interfere with polypeptide accumulation in *E. coli*. First, *E. coli* cells harboring the EgVTE4 or EgVTE4-W290S constructs were supplemented with δ-tocopherol, γ-tocopherol, δ-tocotrienol or γ-tocotrienol after induction of expression. Tocochromanols were extracted and measured by HPLC. While a large proportion of the γ- and δ-tocochromanols were methylated and thereby converted into the corresponding α- and β-tocochromanols in the EgVTE4 expressing cells, no conversion was observed with the EgVTE4-W290S expressing cells (**Figure 4A**). Next, proteins from *E. coli* cells expressing EgVTE4 or EgVTE4-W290S were employed for *in vitro* enzyme assays with SAM and different tocochromanols. The methylation activity of EgVTE4 was highest with γ-tocotrienol and γ-tocopherol, and it was similar for δ-tocopherol and δ-tocotrienol (**Figure 4B**). The EgVTE4-W290S protein was inactive with the different tocochromanols. Therefore, EgVTE4 shows high activity with γ-tocotrienol and γ-tocopherol, but EgVTE4-W290S was inactive, although the two proteins were expressed in *E. coli* at similar levels.

To study the impact of the W to S exchange on the VTE4 activity *in planta*, the full length EgVTE4 and EgVTE4-W290S sequences including the transit peptides were introduced into the *Arabidopsis vte4-1* mutant that is deficient in α-tocopherol but accumulates γ-tocopherol in the leaves (Bergmüller et al., 2003) (**Figure 4C**). The tocochromanol composition in the leaves of the empty vector control (*vte4*+eV) and *vte4*+EgVTE4-W290S plants was similar to the non-transformed *vte4-1* plants. Introduction of EgVTE4 into the *vte4-1* plants resulted in the complementation of α-tocopherol deficiency in the leaves. Therefore, the EgVTE4 protein from Deli x La Mé was capable of converting γ-tocopherol into α-tocopherol in the *Arabidopsis vte4-1* mutant, but the EgVTE4-W290S protein was inactive. Taken together, expression in *E. coli* and in the *Arabidopsis vte4-1* mutant revealed that the W290S substitution in EgVTE4-W290S leads to a non-functional enzyme and therefore is causal for the loss of α-tocopherol and α-tocotrienol in palm tree C59.

### 3.5 Oxidative stability of carotenes is increased in mesocarp of the palm C59

**Figure 5:**
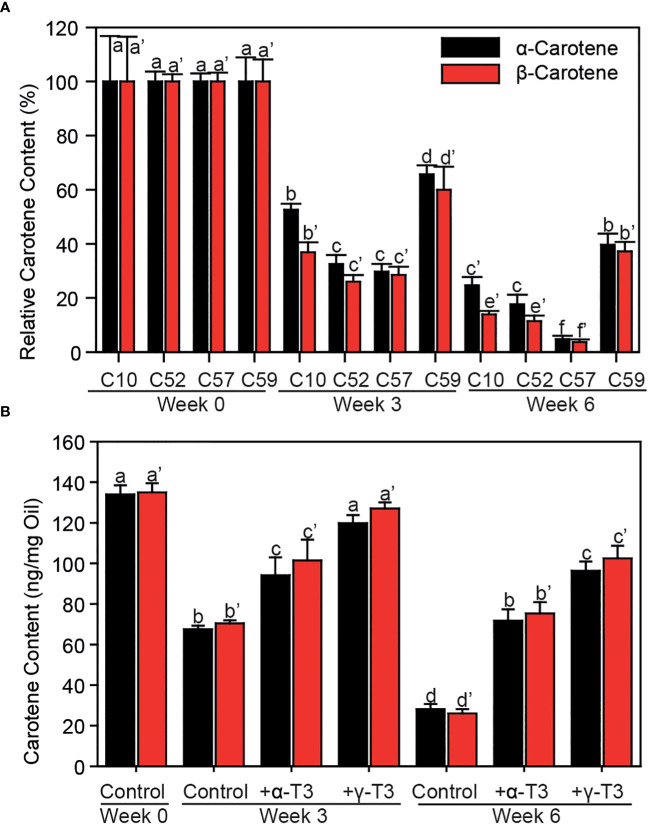
Antioxidant function of tocochromanols during the protection of carotenes in mesocarp and oil against oxidative degradation. **(A)** Lyophilized mesocarp powder of different wild accessions of oil palm fruits was incubated in open tubes at 37°C for different times. Amounts of carotenes are given in % of the initial contents. **(B)** Commercial palm oil was supplemented with different tocochromanols and incubated in open tubes at 37°C for different times. Carotenes were measured by HPLC. One-way ANOVA and Tukey HSD. Mean ± SD; n=3; p<0.01. Letters indicate significant differences (a, b, c for α-carotene; a’, b’, c’ etc. for β-carotene). α-T3, α-tocotrienol; γ-T3, γ-tocotrienol.

To unravel whether the over-accumulation of γ-tocochromanols in palm tree C59 compared with Deli x La Mé contributes to higher oxidative stability of carotenes, the dynamics of carotene degradation were studied. The oxidative stability in mesocarp from palm C59 was compared with other palms (C10, Deli x La Mé; wild lines, C52, C57) which contain similar amounts of tocochromanols, but accumulate α-tocochromanols instead of γ-tocochromanols (**Figure 1**). Homogenized mesocarp samples were incubated under oxidative conditions in open tubes under air for 0, 3 or 6 weeks, followed by carotene analysis by HPLC. After incubation, the contents of α-carotene and β-carotene were declined in all samples (**Figure 5A**). However, the contents of carotenes were more strongly decreased to only 5 to 30% in the three control samples (C10, C52, C57) while carotene amounts stayed elevated in palm C59 (~40% of the content at day 0), indicating that carotene oxidation was mitigated in the mesocarp of palm tree C59.

### 3.6 γ-Tocotrienol exerts elevated antioxidant activity compared with α-tocotrienol in palm oil

Tocochromanols have been described to provide prooxidant activities under certain conditions, in addition to antioxidant activities. Thus, the attenuation of carotene degradation in palm C59 could be due to an increased antioxidant activity of γ-tocotrienol or a reduced prooxidant activity caused by the loss of α-tocotrienol. Therefore, it was important to study the effects of α-tocochromanols and γ-tocochromanols on oxidative stability of the carotenes in palm oil. Commercial palm oil was supplemented with α-tocotrienol or γ-tocotrienol and incubated under oxidative conditions as described above. The supplementation with either of the two tocochromanols efficiently attenuated the degradation of carotene (**Figure 5B**). The γ-tocotrienol supplementation was even more effective than α-tocotrienol in mitigating carotene degradation. Therefore, γ-tocotrienol provides higher antioxidant activity to protect carotenes from oxidation compared with α-tocotrienol. Taken together, these results indicate that the increased oxidative stability of carotenes in palm C59 is due to the superior antioxidant capacity of γ-tocotrienol.

## 4 Discussion

This work identified a wild palm tree that carries a natural polymorphism in the *EgVTE4* gene resulting in an altered tocochromanol profile with improved capacity to protect carotenes against oxidative degradation. The polymorphism in *EgVTE4* of palm tree C59 results in a G to C exchange at position 869 of the open reading frame which causes a W290S exchange on amino acid level. The tryptophan at position 290 is absolutely conserved in all VTE4 sequences from plants, algae and cyanobacteria, and it is even found in other methyltransferases including *Streptomyces coelicolor* GPP-MT. In the GPP-MT sequence, this tryptophan is close to the substrate geranyl-diphosphate and therefore, it is presumably involved in substrate binding. For this reason, it is likely that W290 of EgVTE4 is also in contact with the substrate γ-tocopherol/γ-tocotrienol, and that its replacement with serine in palm tree C59 causes inactivation of the methyltransferase activity. In agreement with this scenario, the EgVTE4 sequence from Deli x La Mé was enzymatically active after expression in *E. coli* or *Arabidopsis*, while the mutant version EgVTE4-W290S was not. The EgVTE4 protein displayed even higher activity with γ-tocotrienol compared with γ-tocopherol in the feeding experiment and *in vitro* assay (**Figures 4A, B**). Additional experiments including determination of kinetic parameters would be required to determine substrate specificity of EgVTE4. Previously, based on a negative correlation between the percentage of tocotrienols and VTE4 activity (% of α forms), EgVTE4 was suggested to convert γ-tocopherol into α-tocopherol more efficiently than γ-tocotrienol into α-tocotrienol (Morcillo et al., 2021). The tree C59 originates from the central region of Cameroon (**Supplementary Table 1**). No other tree with a related trait of low α-tocopherol and low α-tocotrienol contents was identified during the screening of trees from the grooves of different regions in Cameroon. In addition, previous screening programs for tocochromanol content and composition in mesocarp oil which included four varieties from Côte d’Ivoire and 200 trees with different geographic origins (Monde et al., 2009; Luo et al., 2020; Dou et al., 2021), also did not lead to the identification of palm trees with a low α-tocopherol/α-tocotrienol trait. Therefore, the tocochromanol composition of the oil from palm tree C59 is highly unique.

The oxidative degradation of α-carotene and β-carotene in mesocarp of palm tree C59 was attenuated compared with that of the other oil palms C10, C52 and C57 which contain similar total amounts of tocochromanols, but accumulate the α forms instead of the γ forms. The antioxidant function of tocochromanols is well established and they have been shown to stabilize polyunsaturated fatty acids of vegetable oils against oxidation (Kamal-Eldin and Appelqvist, 1996). However, depending on the concentration, some tocochromanols can even show prooxidative effects. For example, α-tocopherol in aqueous mixtures can cause an increased rate of linoleic acid autoxidation (Cillard et al., 1980). In addition, individual tocochromanols differ in their antioxidant capacity during the protection of unsaturated fatty acids. The antioxidant activity of γ-tocopherol is higher than that of α-tocopherol in lard (Olcott and Emerson, 1937). Similarly, γ-tocopherol at low concentrations is a better antioxidant than α-tocopherol in protecting rape seed oil against oxidation (Heinonen et al., 1997). Transgenic cotton seeds, expressing barley homogentisate geranylgeranyl transferase (HGGT) accumulated high amounts of tocotrienols in addition to tocopherols. The antioxidant activity and the oxidative stability in the transgenic seeds was increased demonstrating that high tocochromanol levels protect unsaturated fatty acids in the oil against oxidation (Salimath et al., 2021). Similarly, transgenic soybean seeds, expressing barley HGGT and accumulating polyunsaturated fatty acids, showed an increased antioxidant capacity. However, the oxidative stability index of the seed oil was decreased, presumably because the high accumulation of tocotrienols exerted a prooxidant effect (Konda et al., 2020).

While the antioxidant capacity of tocochromanols to protect fatty acids against oxidative degradation is well established, its impact on preventing carotene degradation has been less well studied. In mineral oil solution incubated at 75°C, protection of β-carotene against oxidation by γ-tocopherol was superior compared with α-tocopherol (Lea and Ward, 1959). During deep frying of palm oil, carotenes were oxidized, and this oxidation was mitigated by supplementation with tocochromanols. Again, the antioxidant effect of γ-tocotrienol was higher than that of α-tocotrienol (Schroeder et al., 2006). The oxidative stability of carotenes during storage in biofortified crops has been identified as a major bottleneck limiting a reliable supply to increase daily intake rates. Transgenic sorghum seeds expressing phytoene synthase to increase carotene amounts, and barley HGGT, accumulated tocotrienols which increased the stability of carotenes during seed storage (Che et al., 2016). Furthermore, transgenic *Arabidopsis* seeds overexpressing, among others, phytoene synthase, led to increased stability of carotenes during seed storage. Co-overexpression with barley HGGT resulted in tocotrienol production and further increased the carotene accumulation and stability (Sun et al., 2021).

Vitamin A deficiency is the leading cause of preventable childhood blindness afflicting half a million children mostly in developing countries every year. Preventive strategies have focused on increasing the provitamin A supply by either nutritional supplements or transgenic bio-fortification of domestic crops. However, transgenic approaches for crop improvement suffer from public acceptance which limits implementation and its impact on combating vitamin A deficiency. Due to the high genetic diversity, wild crop relatives are a valuable resource for crop improvement, and in the case of Oil Palm, represent a nearly untapped treasure trove. The low α-tocochromanol/high γ-tocochromanol trait of palm tree C59 might represent one possible way to improve oxidation stability of the CPO. While γ-tocochromanols *in vitro* exert a higher antioxidant effect compared with α-tocochromanols, α-tocopherol forms show a higher vitamin E activity than γ-tocopherol (Kamal-Eldin and Appelqvist, 1996). This effect is caused by the high affinity of the liver tocopherol binding protein to α-tocopherol which increases the absorption rate of α -tocopherol from the diet (Dutta-Roy et al., 1994). However, vitamin E supply in most countries is much less an issue than vitamin A deficiency. Taken together, the introduction of novel traits for optimized tocochromanol composition or increased total tocochromanol content in CPO into elite oil palm breeding material represents an intriguing strategy to improve oxidation stability and shelf life of CPO.

## 5 Conclusions

Crude palm oil is rich in carotenes (provitamin A) and also contains tocochromanols (vitamin E) as natural antioxidants. However, the content and composition of tocochromanols is insufficient to protect the carotenes against oxidative degradation. It is desirable to obtain a palm oil with highly stable carotene content to fight vitamin A deficiency. The palm tree C59 from Cameroon is unique in that it produces an oil with high γ-tocotrienol instead of α-tocotrienol. The carotenes in the oil from tree C59 display higher oxidative stability compared with other Oil Palm accessions. The tocotrienol trait is based on a polymorphism in the *EgVTE4* gene. As CPO is a major ingredient of many culinary recipes in sub-saharian Africa, this trait can be introgressed into elite Oil Palm material to obtain trees which produce an oil with increased stability of carotenes to protect against vitamin A deficiency.

## Data availability statement

The original contributions presented in the study are included in the article/**Supplementary Material**. Further inquiries can be directed to the corresponding author.

## Author contributions

PG: Conceptualization, Data curation, Formal analysis, Investigation, Methodology, Writing – original draft. DP: Formal analysis, Investigation, Methodology, Writing – review & editing. SS: Investigation, Methodology, Writing – review & editing. KG: Formal analysis, Investigation, Methodology, Writing – review & editing. FD-N: Investigation, Methodology, Resources, Writing – review & editing. REN: Investigation, Methodology, Resources, Writing – review & editing. GN-E: Conceptualization, Methodology, Resources, Supervision, Writing – original draft. PD: Conceptualization, Data curation, Supervision, Writing – original draft, Writing – review & editing.
