# Supplementary material for: Unusual vitamin E profile in the oil of a wild African oil palm tree (Elaeis guineensis Jacq.) enhances oxidative stability of provitamin A
Source: Front Plant Sci. 2024 Jun 27;15:1400852. doi: 10.3389/fpls.2024.1400852 (PMC11236613; doi:10.3389/fpls.2024.1400852)
Supplement: Supplementary file 1 [file DataSheet_1.pdf]

```
Deli X Lamé          1 MTSLL------------------HTCE---P-PS--LRLRSSLHRHSR--LGGRTA---T
XP_010906229 X1     1 MTSLL------------------HTCE---P-PS--LRLRSSLHRHSR--LGGRTA---A
XP_010906230 X2     1 MDAVL------------------RSEH---S-P-----------------FVGMYE---P
Line59               1 MTSLL------------------HTCE---P-PS--LRLRSSLHRHSR--LGGRTA---T
Arabidopsis          1 MKATL------------------AAPS---SLTSLPYRTNSSFGSKSS--LLFRSP---S
Rice                 1 MAHAAAATGALAPLHPLLRCTSRHLCA---S-AS--PRAGLCLHHHRR--R-RRSS---R
Chlamydomonas        1 MPSTA------------------LQGH---TLPS--S--SACLGRATR--HVCRVS---T
Nannochloropsis      1 MKVTL------------------LVWGLLFLPDSVQSFLSPGVHRAAAMNLPGKTRLTST
Nostoc               1 MSAT--------------------------------------------------------
Synechocystis        1 MSTA--------------------------------------------------------

Deli X Lamé         32 --------------PSHLSRPPRSRRLAAARAAMTT--------------AS--------
XP_010906229 X1    32 --------------PSHLSRPPRSRRLAAARAAMTT--------------AS--------
XP_010906230 X2    19 --------------VGYAS-APAARRRAA-KVA-ER--------------GQ--------
Line59              32 -------------- S          L    VA  T--------------AS--------
Arabidopsis         35 --------------SSS-SVSMTTTRGNVAVAAAAT--------------ST--------
Rice                49 --------------RTKLAVRAMAPTLSSSSTA-AA--------------AP--------
Chlamydomonas       31 --------------RSRRAVTVRAGPLETLVKPLTTL------------GKV--------
Nannochloropsis     43 SSGAIQPVRRSLQASSSVVKKPASRLMVNLLTATSTFGASSIIKSLPILGTLVVAGLLAA
Nostoc               5 ------------------------------------------------------------
Synechocystis        5 ------------------------------------------------------------

Deli X Lamé         56 ---DELGK----------GIAEFYDK--SSGLWESIWGDHMHHGFYEAGDPA---TI--T
XP_010906229 X1    56 ---DELGK----------GIAEFYDK--SSGLWESIWGDHMHHGFYEAGDPA---TI--T
XP_010906230 X2    40 ---EELKK----------GIAELYDE--SSEMWEKLWGDHMHHGFYDKDASV---SF--S
Line59              56 ---DELGK----------GIAEFYDK--SSGLWESIWGDHMHHGFYEAGDPA---TI--T
Arabidopsis         58 ---EALRK----------GIAEFYNE--TSGLWEEIWGDHMHHGFYDPDSSV---QLSDS
Rice                72 ---PGLKE----------GIAGLYDE--SSGVWESIWGEHMHHGFYDAGEAA---SM--S
Chlamydomonas       57 ---SDLKV----------GIANFYDE--SSELWENMWGEHMHHGYYPKGAPV-------K
Nannochloropsis    103 RLPGVIARGWRSYNKEENSVGREYDAWTREKILEHYWGEHIHLGYYTEEDQKAGYLK--K
Nostoc               5 -----LYQ----------QIQQFYDA--SSGLWEEIWGEHMHHGYYGADGTE----Q--K
Synechocystis        5 -----LQQ----------QIQQFYDA--SSSLWEQIWGEHMHHGYYGADGKI----K--K

Deli X Lamé         96 GHRAAQIRMVEEALRFAAVSDDP-LKKPKRIVDVGCGIGGSSRYLAKKYGA--KCEGITL
XP_010906229 X1    96 GHRAAQIRMVEEALRFAAVSDDP-LKKPKRIVDVGCGIGGSSRYLAKKYGA--KCEGITL
XP_010906230 X2    80 DHRPAQVRMVQEALAFAGITDDP-LKKPKRIVDVGCGIGGSSRYLAKKYGA--KCEGITL
Line59              96 GHRAAQIRMVEEALRFAAVSDDP-LKKPKRIVDVGCGIGGSSRYLAKKYGA--KCEGITL
Arabidopsis        100 GHKEAQIRMIEESLRFAGVTDEEEEKKIKKVVDVGCGIGGSSRYLASKFGA--ECIGITL
Rice               112 DHRRAQIRMIEESLAFAAVPDDA-EKKPKSVVDVGCGIGGSSRYLANKYGA--QCYGITL
Chlamydomonas       95 SNQQAQIDMIEETLKVAGVT------QAKKMVDVGCGIGGSSRYISRKFGC--TSNGITL
Nannochloropsis    161 NFIGAKYDFIDRMMAFAKLDG-Q-TFKPAKVLDVGCGIGGTTRYIAKKLGTTSQVSGITL
Nostoc              42 NRRQAQIDLIEELLTWAGVQ------TAENILDVGCGIGGSSLYLAGKLNA--KATGITL
Synechocystis       42 ERRQAQIDLIEELLQWSGVQ------QAENILDVGCGIGGSSLYLAQKFNA--KVTGITL

Deli X Lamé        153 SPVQVKRAHALATAEGLEDQVSFQVADALKQPFPDGQFDLVWSMESGEHMPDKTKFVGEL
XP_010906229 X1   153 SPVQVKRAHALATAEGLEDQVSFQVADALKQPFPDGQFDLVWSMESGEHMPDKTKFVGEL
XP_010906230 X2   137 SPVQVKRAHALATAEGLEDQVSFQVADALKQPFPDGQFDLVWSMESGEHMPDKTKFVGEL
Line59             153 SPVQVKRAHALATAEGLEDQVSFQVADALKQPFPDGQFDLVWSMESGEHMPDKTKFVGEL
Arabidopsis        158 SPVQAKRANDLAAAQSLAHKASFQVADALDQPFEDGKFDLVWSMESGEHMPDKAKFVKEL
Rice               169 SPVQAERGNALAAEQGLSDKVSFQVGDALEQPFPDGQFDLVWSMESGEHMPDKRQFVSEL
Chlamydomonas      147 SPKQAARANALSKEQGFGDKLQFQVGDALAQPFEAGAFDLVWSMESGEHMPDKKKFVSEL
Nannochloropsis    219 SQEQVRRAKELAEEQDVTN-AEFQVMDALHMSYPDNSFDLVWACESGEHMPDKKAYVEEM
Nostoc              94 SPVQAARATERAKEAGLSGRSQFLVANAQAMPFDDNSFDLVWSLESGEHMPDKTKFLQEC
Synechocystis       94 SPVQASRAAERATEADLAAEASFQVADAQNMPFADNSFDLVWSLESGEHMPDKTKFMQEC
```

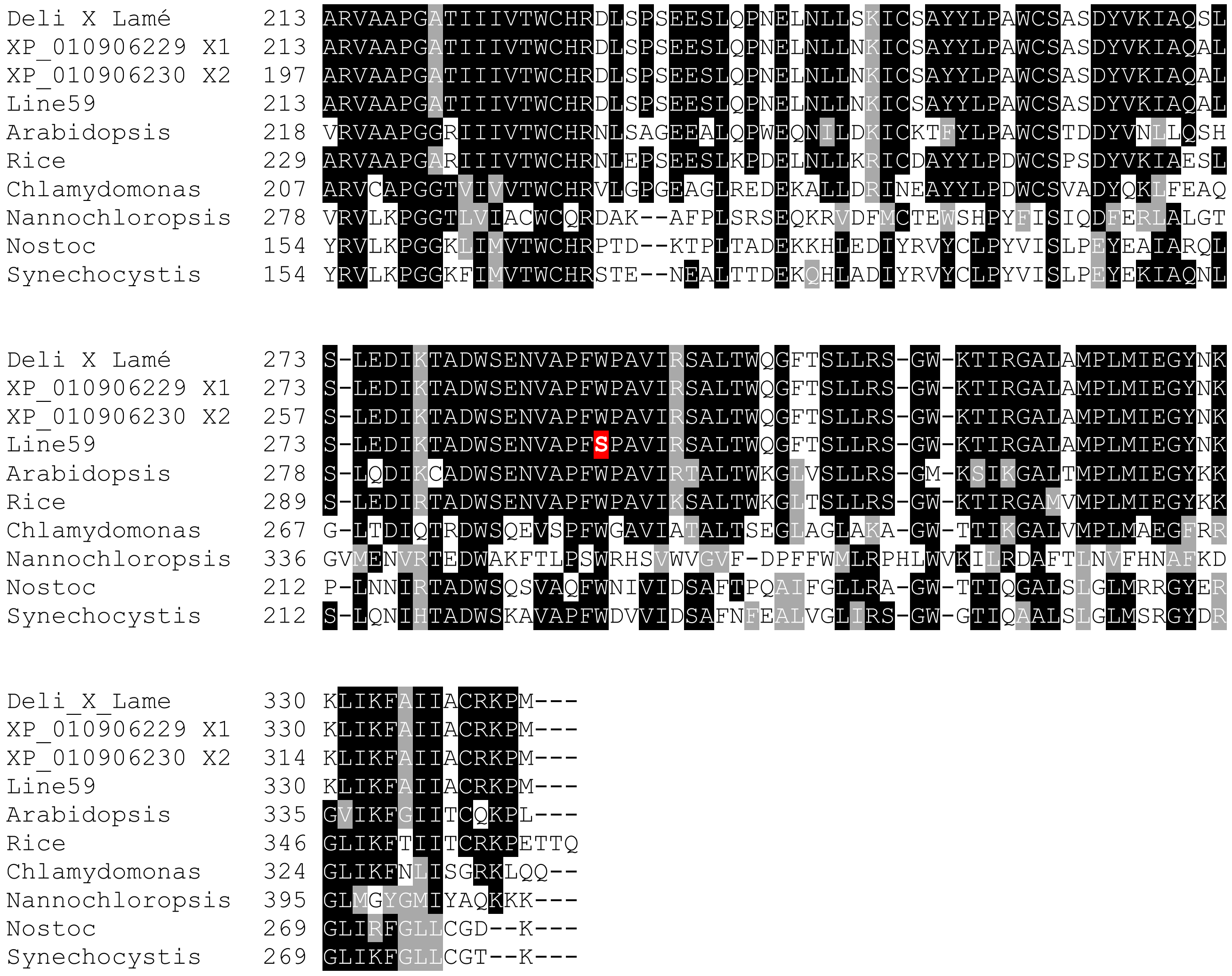

Supplementary Figure 1

Amino acid alignment of VTE4 sequences from different plants, algae and cyanobacteria.

Amino acid sequences were aligned using T-coffee. Black and grey boxes indicate identical and similar amino acids, respectively. The amino acid exchanges in EgVTE4 of palm C59 are highlighted with red boxes. Deli x La Mé, from the rt-PCR product of the EgVTE4 transcript X1 from mesocarp of Deli x La Mé; XP_010906229, from transcript X1 (XP_010907927) of the published oil palm sequence; XP_010906230, from transcript X2 (XP_010907928) of the published oil palm sequence; C59, from the coding region of EgVTE4 transcript X1 of plant C59; *Arabidopsis*, AtVTE4 (Q9ZSK1, At1g64970); Rice, *Oryza sativa* VTE4 (Q6ZIK0); Chlamydomonas, *Chlamydomonas reinhardtii* VTE4 (Q5CB51); Nannochloropsis, *Nannochloropsis gaditana* VTE4 (W7T617); Nostoc, *Nostoc* PCC 7120 γTMT (Q8YW13); Synechocystis, *Synechocystis* PCC 7509 γTMT (WP_009632735).

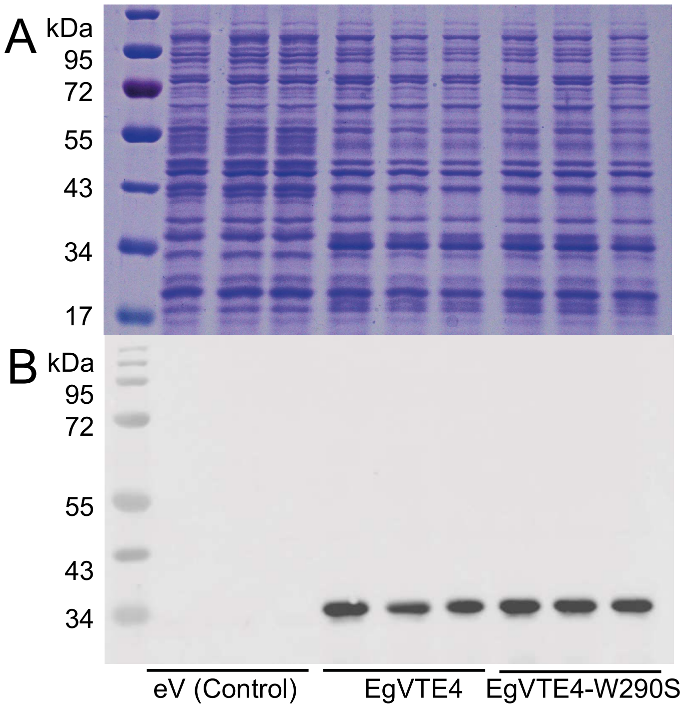

Supplementary Figure 2
Expression of EgVTE4 and EgVTE3-W290S in *E. coli*
(A) The mature form of the EgVTE4 protein lacking the predicted transit peptide and the mutant protein EgVTE4-W290S were expressed in *E. coli* as His-tag fusions. Soluble *E. coli* proteins were separated by SDS polyacrylamide gel electrophoresis and stained with Coomassie Blue. **(B)** After expression in *E. coli* and separation by SDS polyacrylamide gel electrophoresis, His-tagged proteins were detected by Western blotting using the Ni-NTA His detector kit.

Supplementary Table 1

Elite hybrid and wild lines of African Oil Plam (*Elaeis guineensis*) used in this study.

Sample ID	Palm number	Population/ Accession	Region (Cameroon)	Locality	Altitude	Fruit Form
C10	B73.22.26	Dabou Deli x La Mé	Littoral	La Dibamba	47	Tenera
C52	A87.15.2	I16	East	YOKADOUMA	490	Tenera
C57	A87.18.5	I11	North West	BAFUT	1092	Dura
C59	A87.19.3	I69	Center	BIYEM 1	680	Dura

Supplementary Table 2

Oligonucleotides used in this study

Oligo-nucleotide	Sequence (5‘-3‘), (BsaI restriction site, underlined) (6xHis tags, italics)	Comment
Bn3839	GCTCATAAAAGGCCACCGAG	Sequencing LOC105033221 X1 Exon1a forward
Bn3840	TGATAGGCCTTCACGACGAG	Sequencing LOC105033221 X1 Exon1a reverse
Bn3841	TTTCTCTCTCCCTGTGCTCG	Sequencing LOC105033221 X2 Exon1b forward
Bn3842	TGTTTTCCAAGCTGCCATGT	Sequencing LOC105033221 T2 Exon1b reverse
Bn3843	AAGGTGCTGTTTCGGAATGA	Sequencing LOC105033221 Exon 2-4 forward
Bn3844	CCAGGCTGGCAGGTAATATG	Sequencing LOC105033221 Exon 2-4 reverse
Bn3845	GTGATGTTGCTTGGGTGTCA	Sequencing LOC105033221 Exon 4-6 forward
Bn3846	CACCTTGGTCTTTGTTTCTCCA	Sequencing LOC105033221 Exon 4-6 reverse
Bn3895	ATTATTGGTCTCGGCGTATGACCTCTCTCCTCCACACGTG	Cloning full-length EgVTE4 in pBinGG, forward
Bn3896	AATAATGGTCTCCCGTGCTACATAGGTTTACGACAGGC	Cloning full-length/mature EgVTE4 in pBinGG/pTVGG, reverse
Bn3898	AATAATGGTCTCCGGCGAAAATGGTGCCACATTCTCTG	Site directed mutagenesis of full-length/mature EgVTE4 for pBinGG/pTVGG, reverse, 5’ fragment
Bn3899	ATTATTGGTCTCGCGCCTGCAGTTATCCGATCTGCCTTG	Site directed mutagenesis of full-length/mature EgVTE4 in pBinGG/pTVGG forward, 3’ fragment
Bn3902	GTATATTTGGTCTCTGGGTATG*CATCACCATCACCATCAC*ATGACGACGGCGTCTGACGA	Cloning mature EgVTE4 in pTVGG, forward, 6x His
